# Synergistic effects of ground granulated blast furnace slag and nano-silica on the consolidation, compressibility and microstructural behavior of high plasticity clay

**DOI:** 10.1038/s41598-026-37652-2

**Published:** 2026-01-28

**Authors:** Firdevs Uysal, Vedat Yılmaz

**Affiliations:** Department of Civil Engineering, Niğde Ömer Halisdemir University, 51240 Niğde, Turkey

**Keywords:** Soil Stabilization, Consolidation, Compressibility, Swelling, Ground Granulated Blast Furnace Slag-Nano-Silica, Engineering, Materials science

## Abstract

High plasticity clays are prone to excessive deformation and low load-bearing capacity, which frequently leads to structural instability in geotechnical applications. To address these challenges, sustainable and cost-effective stabilization strategies such as the incorporation of nanomaterials and industrial by-products are receiving increasing attention. This study investigates the combined effects of ground granulated blast furnace slag (GGBFS) and nano-silica (NS) on the compressibility, consolidation, and swelling behavior of high plasticity clay using Atterberg limits, standard Proctor compaction, one-dimensional consolidation, and swell tests. Microstructural changes were also investigated using SEM, EDS, and XRD, providing insights into gel-like reaction products and the evolution of soil fabric. Experimental data indicate that the combined use of GGBFS and NS leads to enhanced consolidation-related stiffness (E_oed_), faster consolidation, and reduced swelling in high plasticity clays. The observed improvements in compressibility, consolidation-related stiffness, and swelling behavior are attributed to microstructural refinement induced by stabilization. These effects are associated with pozzolanic reactions, micro-filling, and the formation of gel-like products, as supported by SEM, EDS, and XRD observations. This work demonstrates the integration of GGBFS and NS as a promising strategy for improving the compressibility and swell-related behavior of high plasticity clays.

## Introduction

Highly plastic fine-grained soils, particularly CH and MH clays classified by the USCS, pose geotechnical challenges due to compressibility and volumetric instability^1,2^. These soils are commonly encountered in infrastructure applications, including roadway subgrades, embankment fills, and shallow foundations. Their unfavorable characteristics frequently lead to excessive consolidation settlement, differential deformation, and long-term serviceability problems^3–6^. Moreover, their low hydraulic conductivity can impede pore water dissipation and result in increased pore water pressure under sustained loading, thereby intensifying stability problems^7^. Therefore, the adoption of effective and sustainable ground improvement methods is essential for improving the compressibility, deformation, and serviceability of high plasticity clays^8,9^.

Over the past few decades, chemical stabilization has been widely adopted as an effective method for improving the engineering behavior of high plasticity soils, particularly in terms of consolidation and deformation characteristics^10–13^. Traditional stabilizers such as lime and Portland cement have been extensively studied and applied. However, their production processes are associated with high carbon dioxide emissions, raising significant environmental concerns^14^. In response, employing industrial by-products has gained attention as a more sustainable alternative. Among these, ground granulated blast furnace slag (GGBFS), an industrial residue derived from iron production, offers latent hydraulic properties and significant potential for soil improvement^15^. GGBFS is mainly composed of oxides of calcium, silicon, and aluminum, which facilitate pozzolanic activity when exposed to moisture and alkaline conditions^16,17^. Abdila et al.^18^ showed that adding GGBFS to clayey soils significantly enhances unconfined compressive strength (UCS) and reduces compressibility over various curing periods. These improvements were ascribed to the emergence of pozzolanic reaction products like calcium silicate and aluminate hydrates. Sharma and Sivapullaiah^19^ found that GGBFS-amended fly ash effectively stabilized expansive soils, attributing the strength gains to the synergistic effects of pozzolanic reactions and the formation of cementitious products. Ma et al.^20^ examined the stabilization of water-rich dredged soils using Ca(OH)₂-activated GGBFS and found that incorporating Na₂CO₃ promoted the development of finer capillary structures, thereby enhancing strength and minimizing internal inconsistencies. Collectively, these findings confirm the effectiveness of GGBFS as a sustainable binder in geotechnical applications involving problematic soils. In this context, the present work focuses specifically on consolidation behavior, compressibility, and swelling response.

Advances in nanotechnology have introduced nano-silica (NS) as an effective soil stabilizing agent, primarily due to its exceptional pozzolanic reactivity and expansive specific surface area^21–24^. NS can fill micro-voids, refine the soil fabric, and accelerate the development of binding gels, thereby enhancing the mechanical and consolidation properties of stabilized soils^25,26^. Barbhuiya and Hasan^27^. provided a comprehensive review of NS-stabilized soils and confirmed their efficacy in enhancing pozzolanic activity, improving microstructure, and increasing overall stabilization efficiency. The effectiveness of NS has also been demonstrated in road construction applications, where its combination with cement significantly improved UCS and reduced deformability^28^. Recent studies further corroborate these findings: Kakroudi et al.^29^ reported that NS-treated silty sand exhibited notable improvements in UCS, shear stiffness, and microstructural stability, even under repeated freeze–thaw cycles. Similarly, Ghalandarzadeh et al.^30^ showed that incorporating NS into MICP-treated kaolinite clay significantly enhanced strength, cohesion, and freeze–thaw resistance, underscoring the potential of nano-bio stabilization techniques for fine-grained soils under extreme environmental conditions. In a related study, Ai et al.^24^ confirmed that NS incorporation improved the dynamic strength, deformation stability, and freeze–thaw durability of loess, particularly under cyclic loading, reinforcing its applicability in cold-region geotechnical engineering. While these studies mainly address strength and durability, the present work focuses on consolidation behavior, compressibility, and swelling response.

In the context of soil treatment techniques, the use of multiple additives is often considered to exploit the complementary or synergistic effects of different materials on soil behavior^31–35^. A relevant example is provided by Choobbasti et al.^36^, who found that combining cement and NS in sandy soils improved both maximum dry density and UCS by up to 10%. Similarly, Changizi and Haddad^37^ revealed the effectiveness of utilizing both NS and recycled polyester fiber in significantly enhancing the shear strength, unconfined strength capacity, and stiffness characteristics of cohesive soil, with optimum results observed at 1% NS and 0.3% fiber content, highlighting the synergistic interaction between nanomaterials and synthetic fibers. Kutanaei and Choobbasti^38^ also reported that cemented sand stabilized with both cement and NS exhibited substantial improvements in compressive strength and secant modulus, which they attributed to the synergistic effects of the dual-binder system. Likewise, Oluwatuyi et al.^39^ showed that a 1:1 combination of milled eggshell and cement considerably enhanced the strength and durability of lateritic soils, confirming the viability of agro-industrial synergistic binders in pavement design. In a similar study, Rivera et al.^40^ reported that alkali-activated binders synthesized from fly ash combined with either lime or blast furnace slag significantly enhanced the strength and stiffness of A-4 soils, supporting the application of geopolymer-based dual-binder systems. Furthermore, these findings collectively emphasize the growing recognition of synergistic mechanisms in hybrid soil stabilization strategies. Mehmood et al.^41^ showed that the combined use of microbial carbonate precipitation and calcitic eggshell additive significantly enhanced the strength, cohesion, durability, and permeability resistance of expansive soils compared to individual treatments, highlighting the advantages of integrated bio-mineral stabilization methods. While these studies primarily address strength and durability gains from synergistic stabilization, the present study focuses on consolidation behavior, compressibility, and swelling response.

This research evaluates the influence of varying GGBFS and NS contents on the geotechnical response of high plasticity clay, emphasizing their combined effects on key engineering parameters. The stabilization strategy leverages the latent hydraulic and pozzolanic activity of GGBFS, which is rich in calcium and alumina, together with the high surface area and pozzolanic reactivity of NS, which promotes gel formation and refines the soil microstructure. While the individual effects of GGBFS and NS on soil stabilization have been extensively documented in the literature, their combined influence on the consolidation behavior of high plasticity clays has not been sufficiently explored. Existing studies have predominantly focused on strength-related performance, whereas the implications of GGBFS–NS interaction on one-dimensional consolidation response, compressibility indices, and consolidation-related stiffness remain unclear. To address this gap, the present study investigates the synergistic effects of GGBFS and NS on the consolidation behavior, compressibility, and swelling response of a high plasticity clay. This investigation is based on an experimental program including Atterberg limits, compaction, one-dimensional consolidation, and swell tests. Consolidation-related parameters (C_c_, C_s_, c_v_, m_v_, and E_oed_) are evaluated and interpreted in conjunction with complementary microstructural analyses (SEM, EDS, and XRD), providing a consolidation-focused interpretation of dual-binder stabilization mechanisms. The findings highlight the potential of GGBFS–NS dual-binder systems as a sustainable and efficient strategy for improving the performance of fine-grained soils.

## Materials and methods

### Materials

This research examines the effects of GGBFS and NS on the engineering, consolidation, swelling, and microstructural behavior of a laboratory-prepared high plasticity clay (LPHPC), which was specifically formulated to simulate expansive soil conditions. The LPHPC was prepared by mixing 25% Na-bentonite and 75% kaolinite (by dry weight). This laboratory-prepared high-plasticity clay was used to ensure a homogeneous and reproducible soil system under controlled laboratory conditions.

**Fig. 1:**
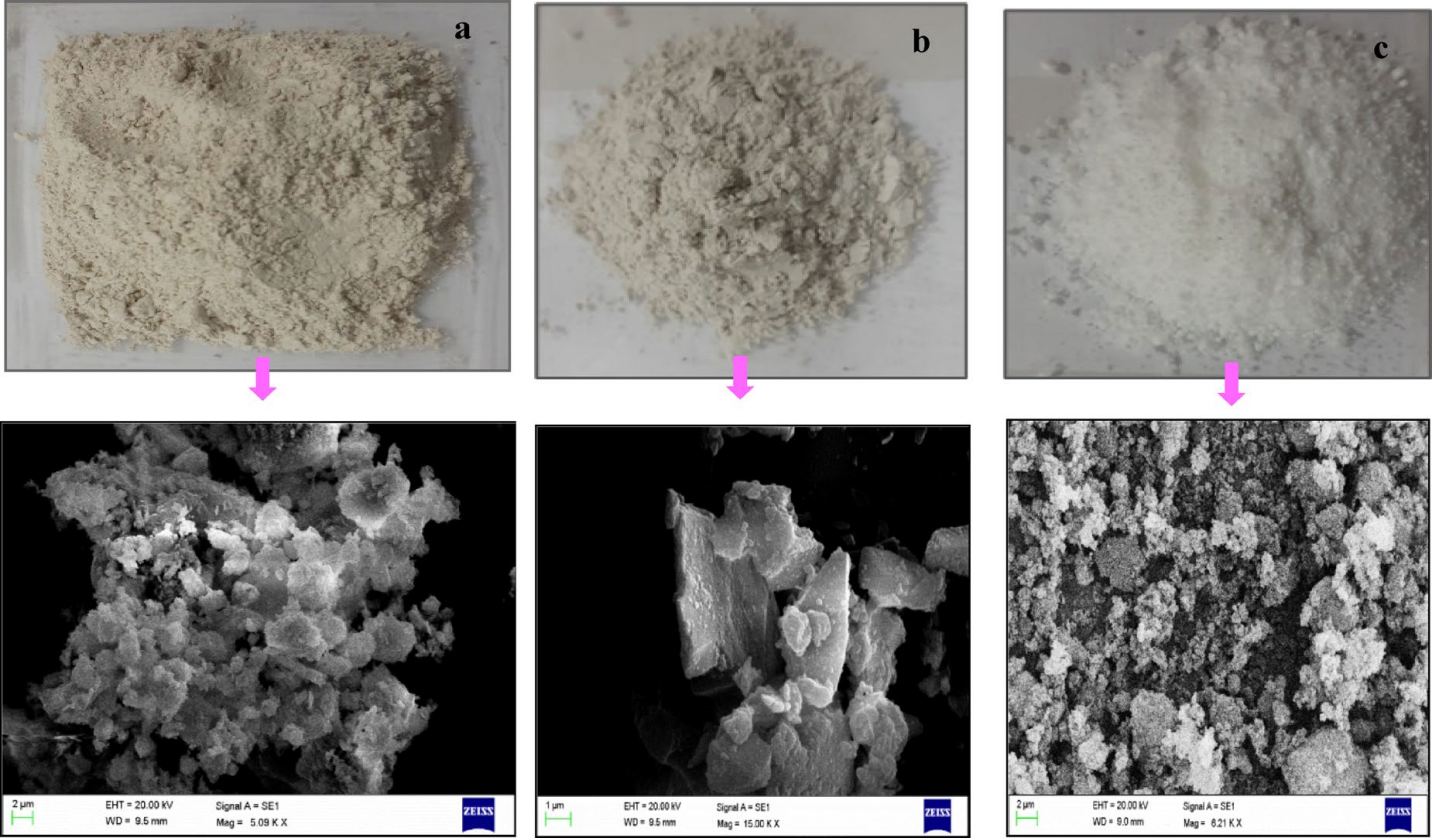
SEM images of the materials: (**a**) LPHPC, (**b**) GGBFS, (**c**) NS.

**Fig. 2:**
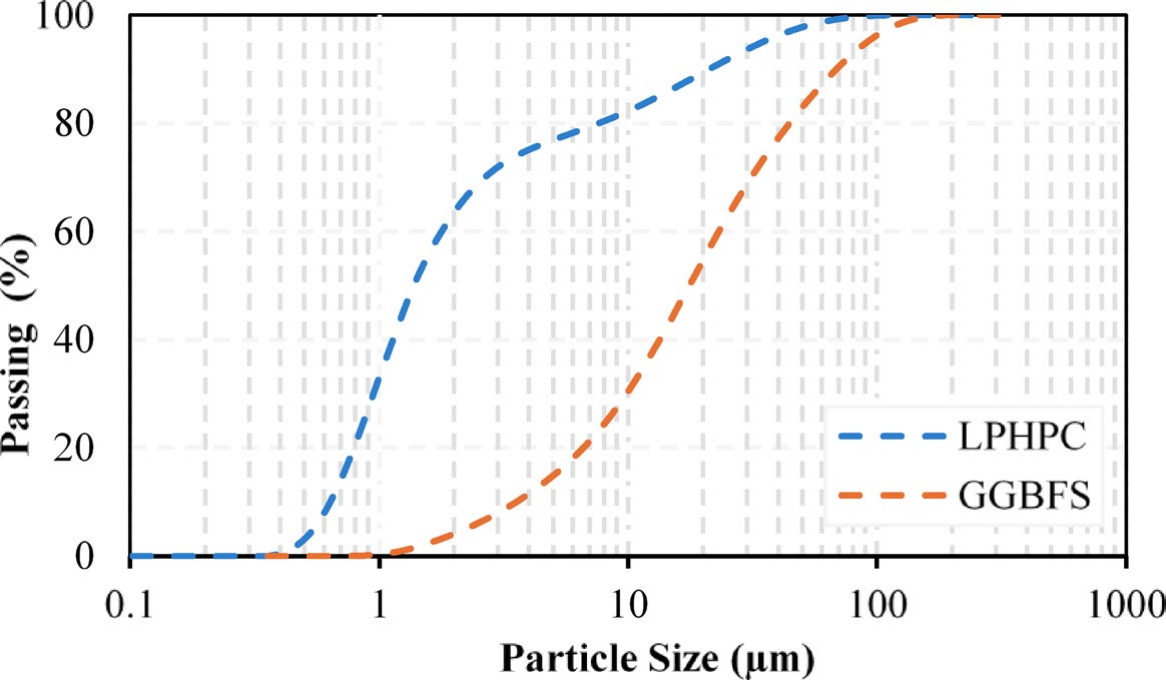
Particle size distribution of LPHPC and GGBFS.

The use of engineered or reconstituted soil mixtures is a common methodological approach in soil stabilization studies to isolate soil–binder interactions and minimize the effects of natural variability in mineralogy, organic matter, and pore-water chemistry. For example, Seif et al.^42^ combined sand and clay fractions to achieve a controlled balance between strength and impermeability, facilitating a mechanistic assessment of NS-induced soil behavior. Similarly, Latha and Murugesan^43^ emphasized that the performance of stabilized earth materials strongly depends on the controlled selection of soil fractions, rather than strict replication of natural soil deposits. In this context, the laboratory-prepared clay serves as a representative model system for high-plasticity clay behavior. However, it is acknowledged that natural expansive clays with mixed mineralogy, organic matter, and soluble salts may exhibit more complex responses. Figure 1 presents the SEM micrographs of the materials used in this study, including LPHPC, GGBFS and NS, while Fig. 2 presents the particle size distribution of LPHPC and GGBFS. Atterberg limit tests revealed a liquid limit (LL) of 106.61%, a plastic limit (PL) of 24.13%, and a resulting plasticity index (PI) of 82.48%. Based on the USCS classification, the material is classified as CH, characterizing it as a high plasticity clay with pronounced swelling behavior. GGBFS, an industrial by-product of the steel manufacturing process, was utilized as the primary stabilizing agent in this study. It is characterized by high contents of calcium oxide (CaO), silicon dioxide (SiO₂), and aluminum oxide (Al₂O₃), which facilitate the generation of cementitious compounds through latent hydraulic and pozzolanic reactions upon exposure to water.

The GGBFS used exhibited a specific gravity of 2.85 and a specific surface area of 1.266 m^2^/g, as measured using the multi-point BET method, indicating a finely ground material with enhanced reactivity. The chemical compositions of Na-bentonite, kaolinite, LPHPC and the GGBFS are presented in Table 1. To further improve the early stage pozzolanic reactivity and microstructural refinement of the treated soil, NS was incorporated as a secondary additive. The NS utilized in this study was an amorphous, fumed silica powder with a particle size distribution between 15 and 35 nm and purity exceeding 99.5%. The material exhibited an exceptionally high specific surface area, ranging from 150 to 550 m^2^/g, which is known to enhance pozzolanic activity and promote the development of calcium-rich gel-like reaction products within stabilized soil matrices.

**Table 1:** The chemical compositions of Na-bentonite, kaolinite, LPHPC and the GGBFS.

Oxide (%)	SiO_2_	CaO	Al_2_O_3_	Fe_2_O_3_	MgO	K_2_O	Na_2_O	SO_3_	Mn_3_O_4_	TiO_2_	LOI*
Na-Bentonite	64.704	4.299	18.764	4.892	2.221	1.362	2.764	0.047	0.125	0.444	0.378
Kaolinite	80.612	1.837	15.530	0.624	0.305	0.390	0.060	0.155	0.004	0.292	0.191
LPHPC	77.186	2.504	15.928	1.646	0.715	0.647	0.668	0.142	0.039	0.337	0.188
GGBFS	36.824	38.834	13.314	0.727	5.648	0.777	–	0.562	1.87	0.761	0.683

*LOI: Loss on ignition.

**Fig. 3:**
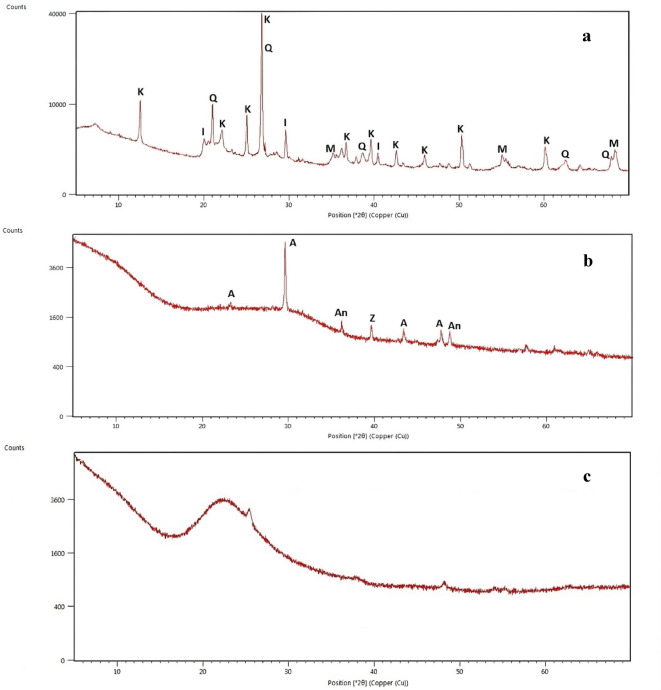
XRD patterns of LPHPC (**a**), GGBFS (**b**), and NS (**c**). K, kaolinite; Q, quartz; I, illite; M, montmorillonite; A, albite; An, anorthite; Z, zeolite.

The true density of the NS was measured to be approximately 2.2 g/cm^3^, while its bulk density remained below 0.1 g/cm^3^, indicating a highly porous, low-mass structure conducive to uniform dispersion. The chemical composition of NS is presented in Table 2. In addition to chemical characterization, the mineralogical profiles of LPHPC, GGBFS, and NS were examined via X-ray diffraction (XRD) analysis, and the corresponding diffraction patterns are shown in Fig. 3. According to the XRD results, the untreated LPHPC exhibits characteristic reflections of kaolinite (K), quartz (Q), illite (I), and montmorillonite (M), confirming the presence of typical clay minerals (Fig. 3a). The XRD pattern of GGBFS exhibits a predominantly amorphous structure with minor crystalline phases such as albite (A), anorthite (An), and zeolitic phases (Z) (Fig. 3b). The XRD pattern of NS is characterized by an amorphous halo with no distinct crystalline peaks (Fig. 3c).

**Table 2:** The chemical compositions of NS.

Material	Result (%)
Si	> 97
Fe	0.002
Ca	0.007
Ti	0.012
Na	0.003
Others	< 0.01

### Test methods

#### Mixture design and sample preparation

The mixture design adopted in this study was developed to evaluate the individual and synergistic effects of GGBFS and NS on the consolidation behavior of a high plasticity clay, as presented in Table 3. In this study, synergy is defined as the additional performance gain observed in dual-binder systems compared to mixtures stabilized with GGBFS alone. Within this framework, GGBFS and NS were deliberately selected due to their distinct pozzolanic and latent hydraulic characteristics, allowing the investigation of stabilization mechanisms at both the micro- and macro-scales. GGBFS was employed as the primary stabilizing agent due to its latent hydraulic reactivity and well-documented efficiency in enhancing soil performance. Upon hydration, it promotes the development of cementitious gel-like reaction products that contribute to reduced compressibility and enhanced consolidation-related stiffness in clayey soils. Four percentages by dry weight of soil (10%, 20%, 30%, and 40%) were selected based on prior optimization studies and relevant literature findings^44,45^. This range covers GGBFS contents widely reported as effective for fine-grained soil stabilization, while avoiding excessively high binder contents that may adversely affect workability, compaction efficiency, and material economy. Previous studies indicate that GGBFS contents beyond approximately 40% provide only limited additional improvements relative to the increased binder demand. NS was incorporated as a secondary additive due to its exceptionally high specific surface area and strong pozzolanic reactivity, particularly at low concentrations. Based on previous research, NS contents of 1% and 1.5% by dry weight of soil were selected^21,24,46^. Low NS contents have been reported to be sufficient to enhance micro-filling effects and pozzolanic reactivity, whereas higher contents may promote particle agglomeration and increased water demand. In addition, preliminary observations during mixture preparation indicated that NS contents above 1.5% reduced workability and hindered uniform compaction. The inclusion of NS was intended to accelerate GGBFS-induced pozzolanic reactions and enhance the soil microstructure by filling micro-pores, thereby reducing the void ratio, improving compressibility, and lowering the settlement potential.

**Table 3:** Mixture composition of laboratory-prepared soil samples.

Mix Code	LPHPC (%)	GGBFS (%)	NS (%)
Control	100	0	0
1	90	10	0
2	80	20	0
3	70	30	0
4	60	40	0
5	90	10	1
6	80	20	1
7	70	30	1
8	60	40	1
9	90	10	1.5
10	80	20	1.5
11	70	30	1.5
12	60	40	1.5

**Fig. 4:**
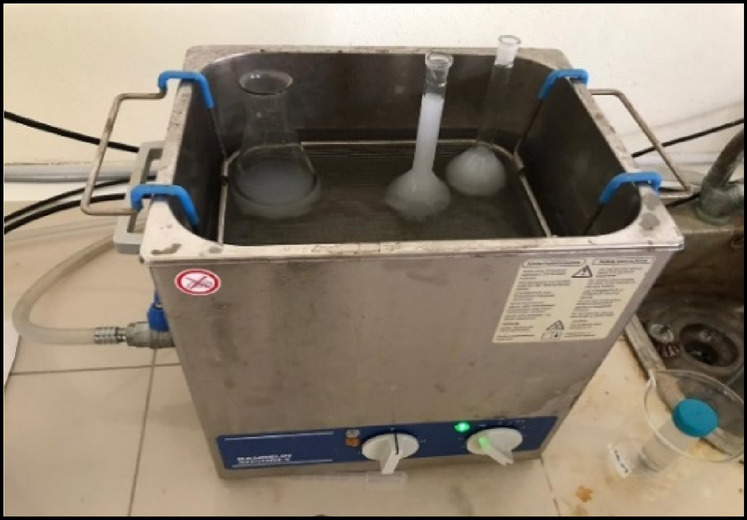
Ultrasonic dispersion of NS in deionized water.

In this study, a synthetic high plasticity clay (LPHPC) was produced entirely under laboratory conditions using commercially available sodium bentonite (25%) and kaolinite (75%), which were sourced from local suppliers, to reproduce the characteristic swelling behavior of expansive soils, rather than using a field-collected soil. All mixtures were prepared in accordance with the proportions defined in Table 3, incorporating various percentages by dry weight of GGBFS and NS. Initially, the dry components (LPHPC and GGBFS) were thoroughly mixed to ensure uniform distribution. For binary mixtures containing NS, the additive was pre-dispersed in deionized water using an ultrasonic bath in order to minimize agglomeration and facilitate uniform integration within the soil matrix (Fig. 4). The ultrasonic bath facilitated the breakdown of particle agglomerates and enhanced the dispersion of NS, thereby ensuring effective integration into the soil matrix and maximizing its pozzolanic reactivity. The resulting suspension was gradually added to the dry soil blend and mixed thoroughly. The prepared mixtures were subsequently utilized in a series of laboratory tests, including Atterberg limits, compaction, one-dimensional consolidation, swell potential assessment, and microstructural analyses (SEM, EDS, and XRD), in alignment with the objectives of the experimental program.

#### Consistency and compaction tests

Consistency and compaction characteristics of the untreated and stabilized LPHPC were evaluated through Atterberg limit and standard Proctor compaction tests. These tests aimed to assess the influence of GGBFS and NS on the plasticity and compactability of the soil mixtures, providing insights into their suitability for geotechnical applications. Atterberg limit tests were performed to determine the liquid limit (LL), plastic limit (PL), and plasticity index (PI) of both untreated and treated LPHPC samples containing various proportions of GGBFS and NS. The liquid limit was determined using the cone penetration method in accordance with BS 1377–2^47^ while the plastic limit was evaluated using the manual thread-rolling method following ASTM D4318^48^. The stabilization strategy included GGBFS at 10%, 20%, 30%, and 40% by dry weight of soil, combined with NS at 1% and 1.5% at each GGBFS level. Standard Proctor compaction tests were conducted in accordance with ASTM D698^49^ to evaluate the compaction behavior of LPHPC mixtures stabilized with GGBFS and NS. The primary objective was twofold: first, to investigate the influence of stabilization on soil compatibility; and second, to determine the optimum moisture content (OMC) and maximum dry unit weight (γ_d,max_) for each mixture to be used in subsequent consolidation tests. The same stabilization combinations (i.e., GGBFS at 10–40% and NS at 1% and 1.5%) were applied in these tests by dry weight of soil.

#### One-dimensional consolidation test

**Fig. 5:**
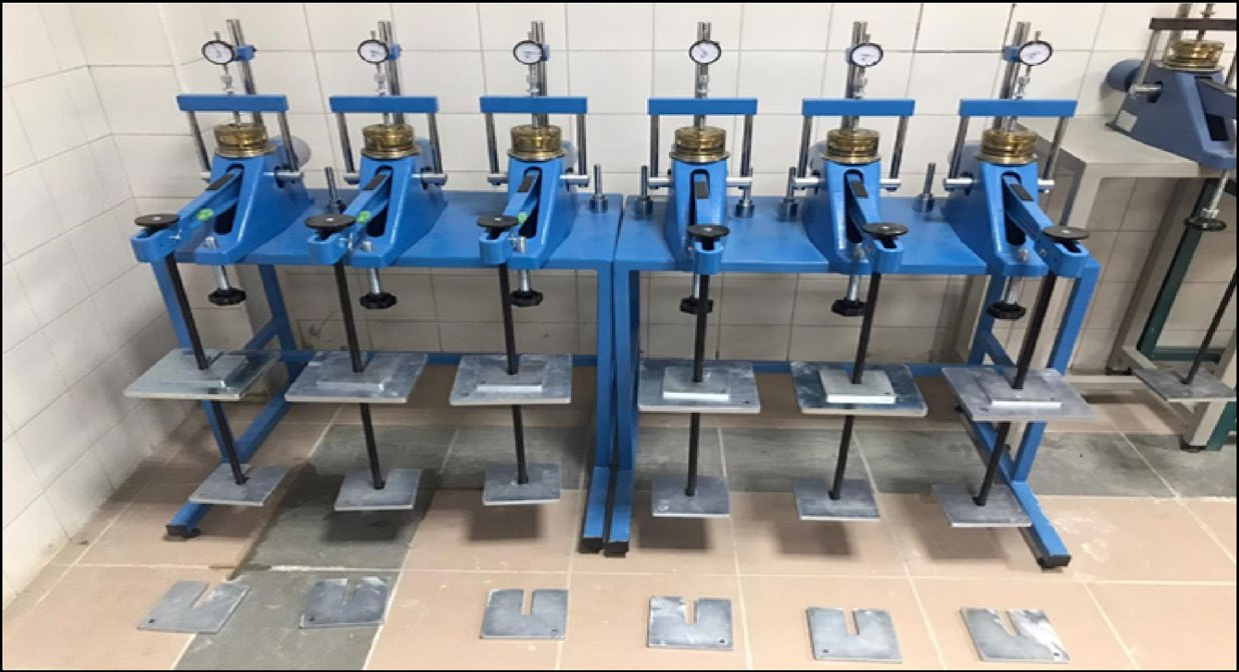
Consolidation test setup and specimen preparation process.

One-dimensional consolidation tests were conducted to assess the compressibility behavior and settlement characteristics of untreated and stabilized LPHPC samples (Fig. 5). The tests were carried out in accordance with ASTM D2435^50^ The primary aim was to examine how GGBFS and NS additives influence the rate and magnitude of primary consolidation, as well as the overall stiffness of the treated soil matrix.

The test also provided key parameters such as the compression index (C_c_), recompression index (C_s_), coefficient of consolidation (c_v_), and coefficient of volume compressibility (m_v_), which are essential for predicting long-term settlement behavior in geotechnical design. Mixtures were prepared with GGBFS contents of 10%, 20%, 30%, and 40%, and NS at 1% and 1.5% by dry weight of soil. All specimens were compacted at their respective optimum moisture content (OMC) and maximum dry unit weight (γ_d,max_) values as determined from the Proctor tests.

#### Swell test

The expansive behavior of the LPHPC, in both untreated and stabilized forms with various additive combinations, was examined through one-dimensional swell tests conducted using a standard oedometer apparatus, following the procedures outlined in ASTM D4546, Method A^51^. The test is widely used to evaluate the vertical deformation behavior of fine-grained soils subjected to inundation under low surcharge, replicating the field conditions of expansive soils. Remolded LPHPC specimens were prepared at their respective optimum moisture contents, determined from Standard Proctor compaction tests, and then placed into oedometer rings measuring 50 mm in diameter and 20 mm in height. A vertical seating pressure of 7 kPa was applied to ensure uniform initial contact. Following specimen placement, distilled water was gradually introduced into the cell to initiate swelling under free-swell conditions.

## Results and discussion

This section presents and interprets the experimental findings obtained from the evaluation of LPHPC samples stabilized with varying proportions of GGBFS and NS. The results are organized into four key categories: consistency and compaction characteristics, one-dimensional consolidation behavior, swell potential, and microstructural features. The experimental results were examined in detail and compared with findings from previous studies to emphasize the effectiveness of the adopted stabilization approach.

### Consistency and compaction tests

**Fig. 6:**
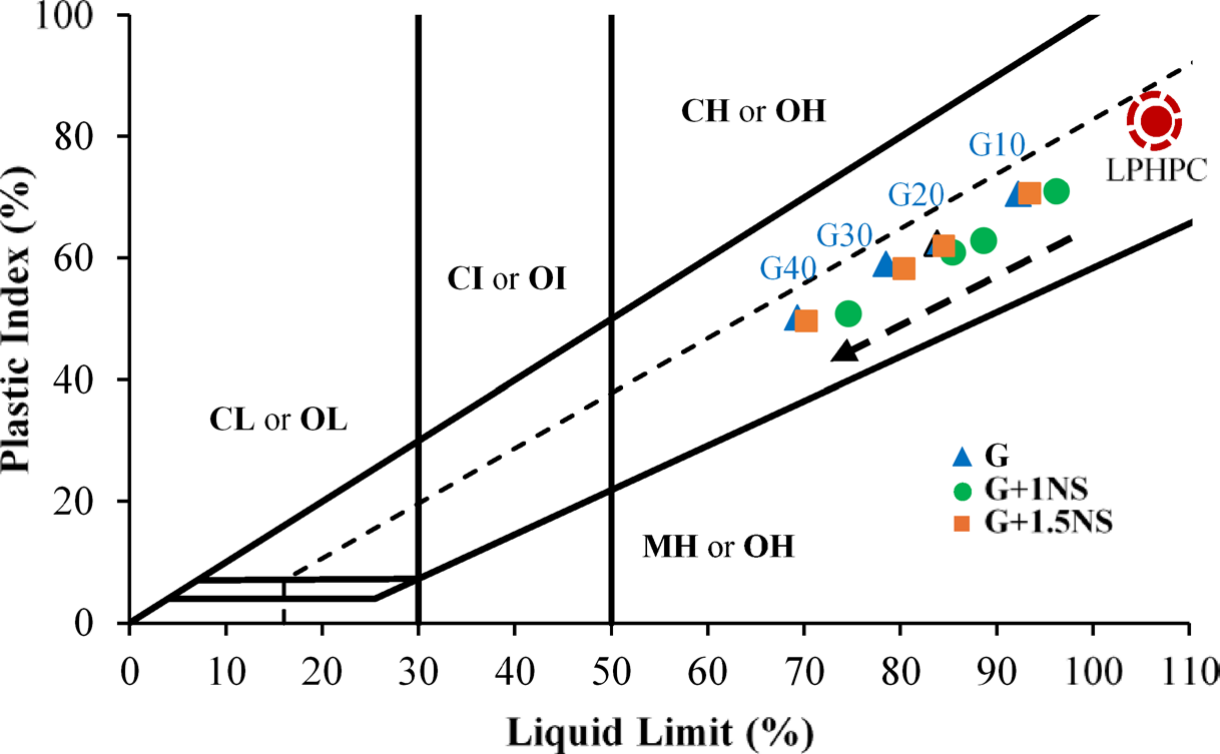
Effect of GGBFS and NS on the plasticity classification of LPHPC mixtures.

The initial position of the LPHPC sample on the plasticity chart indicates a notably high liquid limit (LL = 106.61%) and plasticity index (PI = 82.48%), classifying the untreated soil as CH (high plasticity clay) according to the USCS (Fig. 6). This classification refers to a soil type with high water sensitivity and deformation potential, typically associated with expansive behavior in geotechnical applications. Upon stabilization with varying proportions of GGBFS (10%, 20%, 30%, and 40%) and NS (1% and 1.5%), a consistent shift in classification boundaries is observed, as illustrated in Fig. 6. In the figure, ‘G10’, ‘G20’, ‘G30’, and ‘G40’ represent 10%, 20%, 30%, and 40% GGBFS content, respectively, while ‘G + 1NS’ and ‘G + 1.5NS’ indicate the incorporation of 1% and 1.5% NS at each GGBFS level. Samples treated with GGBFS in the range of 10% to 40% demonstrate a gradual decline in both LL and PI, with their positions shifting downward and leftward on the plasticity chart. This behavior is mainly due to the filler effect of GGBFS and the formation of cementitious compounds that enhance matrix densification^52,53^. The incorporation of NS at 1% and 1.5% induces a slightly different trend. PI values decrease relative to GGBFS-only mixtures, whereas LL values remain relatively elevated. This response is likely related to the high surface area and moisture affinity of NS, which can alter the water distribution within the soil matrix and delay the reduction in consistency limits. Similar observations have been reported by Kalhor et al.^54^, who noted that NS incorporation influenced Atterberg limits and enhanced soil microstructure by altering moisture behavior. Despite this, the overall movement of data points remains toward lower plasticity regions, signifying a partial transition from CH to MH/CL zones, and indicating improved material consistency and reduced workability challenges. Collectively, these trends suggest that GGBFS, either alone or in combination with NS, is effective in reducing the plasticity of high plasticity clays. Nevertheless, the persistence of moderately high LL and PI values in most stabilized samples indicates that the treated clay remains susceptible to moisture variations. Therefore, further optimization of GGBFS and NS mix ratios may be required to ensure reliable performance in moisture-sensitive engineering applications.

**Fig. 7:**
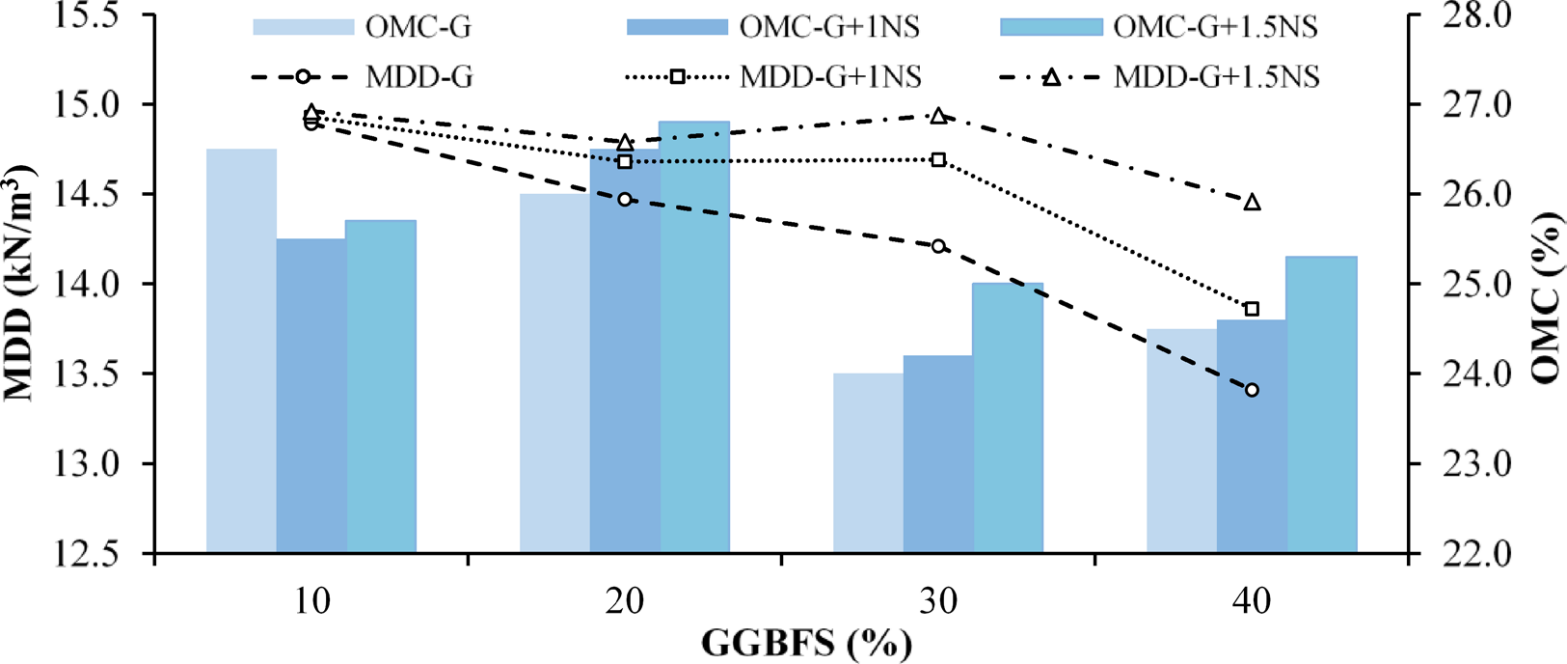
MDD and OMC of LPHPC stabilized with GGBFS and NS.

Standard Proctor tests were conducted with two primary objectives. The first was to evaluate the effects of GGBFS and NS on the compaction behavior of fine-grained soil in the context of stabilization. The second objective was to determine the optimum moisture content (OMC) and maximum dry density (MDD) values for each mixture, which were subsequently used in the preparation of one-dimensional consolidation test specimens. For the first objective, compaction tests were performed on LPHPC mixtures prepared with varying GGBFS contents (10%, 20%, 30%, and 40% by dry weight) to evaluate its influence on compaction behavior. Subsequently, NS was incorporated into the GGBFS treated mixtures at contents of 1% and 1.5% (by dry weight of soil) to evaluate the changes in MDD and OMC resulting from its addition. A gradual decrease in MDD was observed with increasing GGBFS content. This behavior is attributed to changes in the particle-size distribution and packing characteristics of the soil skeleton induced by slag incorporation. These changes alter compaction efficiency under a given compactive effort and are consistent with the observed MDD–OMC trends shown in Fig. 7. However, the incorporation of NS at 1% and 1.5% consistently enhanced MDD values across all GGBFS levels, with the most pronounced improvement observed at 20% GGBFS and 1.5% NS. This trend highlights the beneficial role of NS in promoting matrix densification through micro-filling and partial improvement in particle packing. Nevertheless, at the highest GGBFS content (40%), the addition of 1.5% NS resulted in only limited, and in some cases slightly reduced, gains in MDD. This behavior can be attributed to the accumulation of ultra-fine particles from both GGBFS and NS, which increases the specific surface area and water demand. As a result, particle agglomeration is promoted. These effects may hinder uniform dispersion, alter pore-size distribution through premature pore filling, and limit effective particle rearrangement during compaction, thereby reducing compaction efficiency. Overall, these observations suggest the existence of a practical upper threshold for nanomaterial content in soil stabilization applications.

### One-dimensional consolidation test

The compressibility behavior and settlement potential of high plasticity clay stabilized with GGBFS and NS were evaluated through a series of one-dimensional consolidation tests. The experimental program included samples treated with four different GGBFS contents (10%, 20%, 30%, and 40% by dry weight), both individually and in combination with NS at 1% and 1.5%. The tests were conducted under five loading stages (25, 50, 100, 200, and 400 kPa) and two unloading stages (200 and 100 kPa) each maintained for 24 h. In this context, 400 kPa was selected as a representative stress level for detailed comparison, as it corresponds to a medium-to-high effective stress range relevant to typical subgrade and foundation conditions.

For practical settlement assessment, C_c_, C_s_, c_v_ and m_v_ are considered the most critical parameters, as they are directly used in consolidation settlement and time-rate calculations. Accordingly, this sequence has been followed in the present study, with the consolidation parameters discussed in relation to their practical relevance, while the remaining parameters are included to provide complementary insight.

**Fig. 8:**
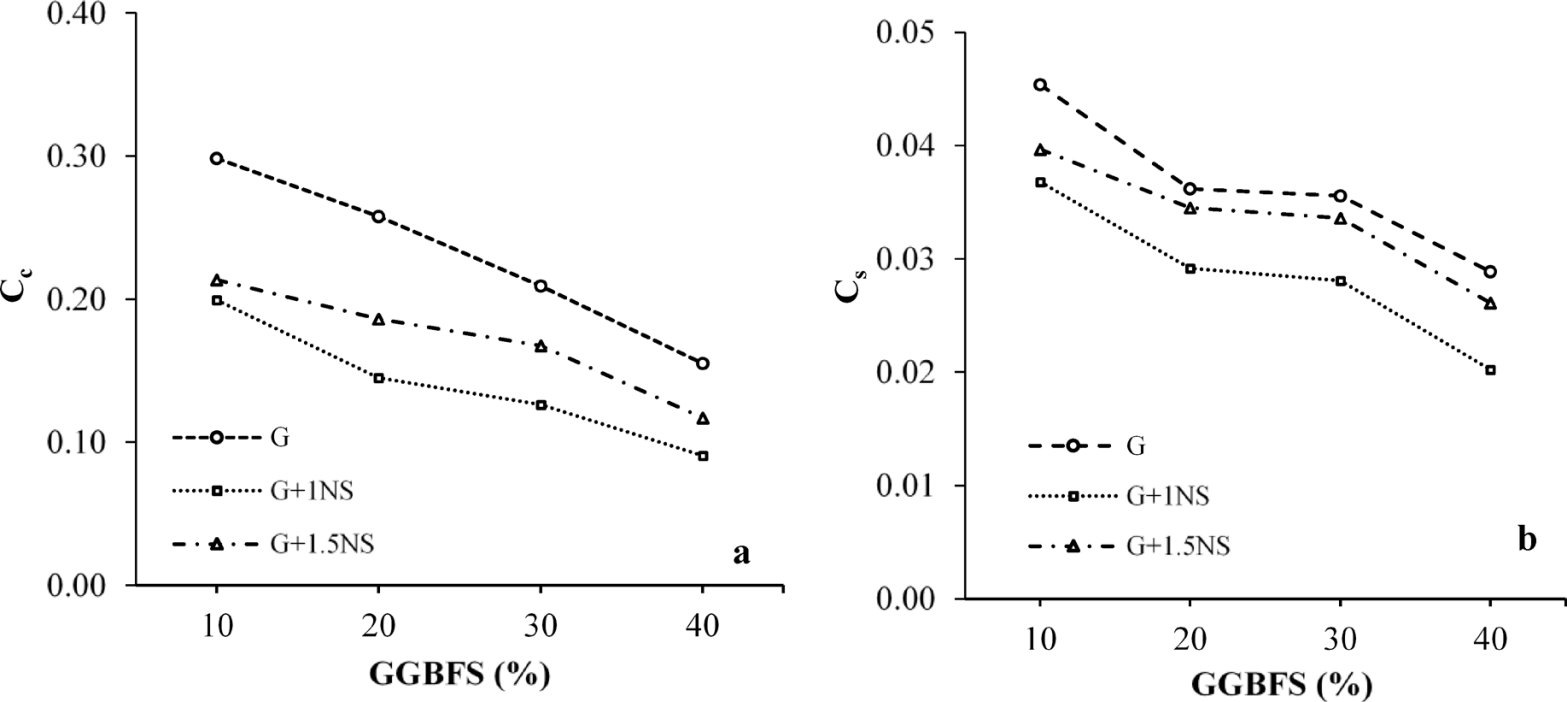
Variation in (**a**) C_c_ and (**b**) C_s_ of LPHPC stabilized with GGBFS and NS.

The C_c_, a key parameter reflecting the magnitude of primary consolidation settlement under applied stress, was measured as 0.308 for untreated LPHPC, indicating a highly compressible nature typical of expansive clays. As illustrated in Fig. 8a, C_c_ consistently decreased with increasing GGBFS content, demonstrating the effectiveness of GGBFS in reducing soil compressibility. Similar results were reported by Zhu et al.^55^, who observed a significant reduction in the C_c_ of GGBFS-stabilized dredged clay, confirming the reliability of slag-based stabilization in fine-grained soils. The incorporation of NS in combination with GGBFS was found to further enhance the reduction in the C_c_, indicating a synergistic amplification effect compared to GGBFS-based stabilization alone. The inclusion of 1% NS yielded the lowest C_c_ values across all GGBFS levels, with the most pronounced improvement observed at 40% GGBFS, where C_c_ dropped to approximately 0.09 (Fig. 8a). When the corresponding mixtures are compared, the incorporation of 1% NS into the 40% GGBFS-stabilized mixture is associated with an additional reduction of approximately 42% in the C_c_ relative to the mixture stabilized with GGBFS alone. This outcome suggests that NS contributes to microstructural densification through micro-filling effects and enhanced pozzolanic reactivity. Although 1.5% NS also contributed to the reduction in C_c_, its effect was slightly less pronounced, likely due to an excess of fine particles disrupting optimal packing (Fig. 8a). These results indicate that a 1% NS provides a more balanced and efficient contribution to compressibility reduction, especially when combined with an optimal GGBFS content.

C_s_ is a key parameter that reflects the soil’s tendency to undergo volumetric expansion upon moisture intake. In untreated LPHPC mixtures, the C_s_ value was determined to be 0.062, indicating a relatively high swelling potential. As shown in Fig. 8b, C_s_ consistently decreased with increasing GGBFS content, suggesting that the incorporation of GGBFS effectively reduces the expansive behavior of the soil. These findings confirm that GGBFS contributes to reduced swelling deformation and improved volumetric stability of the treated LPHPC, particularly after loading. This improvement underscores the positive role of GGBFS in increasing the soil’s resistance to volume change. The reduction in C_s_ with increasing GGBFS content can be attributed to the development of calcium-enriched gel-like reaction products, as inferred from SEM–EDS elemental distributions, which contribute to microstructural densification. These gels fill pore spaces and contribute to microstructural stabilization, thereby reducing post-unloading swelling, as reflected by the lower C_s_ values. The addition of NS to GGBFS-stabilized LPHPC mixtures led to a further reduction in the C_s_ across all GGBFS levels. As illustrated in Fig. 8b, the incorporation of 1% NS caused a more significant decrease in C_s_ values compared to mixtures containing GGBFS alone. This improvement can be attributed to the high specific surface area and pozzolanic reactivity of NS, which facilitates additional cementitious gel formation and enhances inter-particle bonding. Mixtures containing 1.5% NS showed slightly higher C_s_ values than those with 1% NS, especially at lower GGBFS contents. This may be due to an excessive amount of fine particles, which may disrupt optimal packing and affect water distribution. These findings suggest that 1% NS provides a more effective and balanced contribution to swelling reduction, particularly when combined with moderate-to-high GGBFS levels (Fig. 8b).

**Fig. 9:**
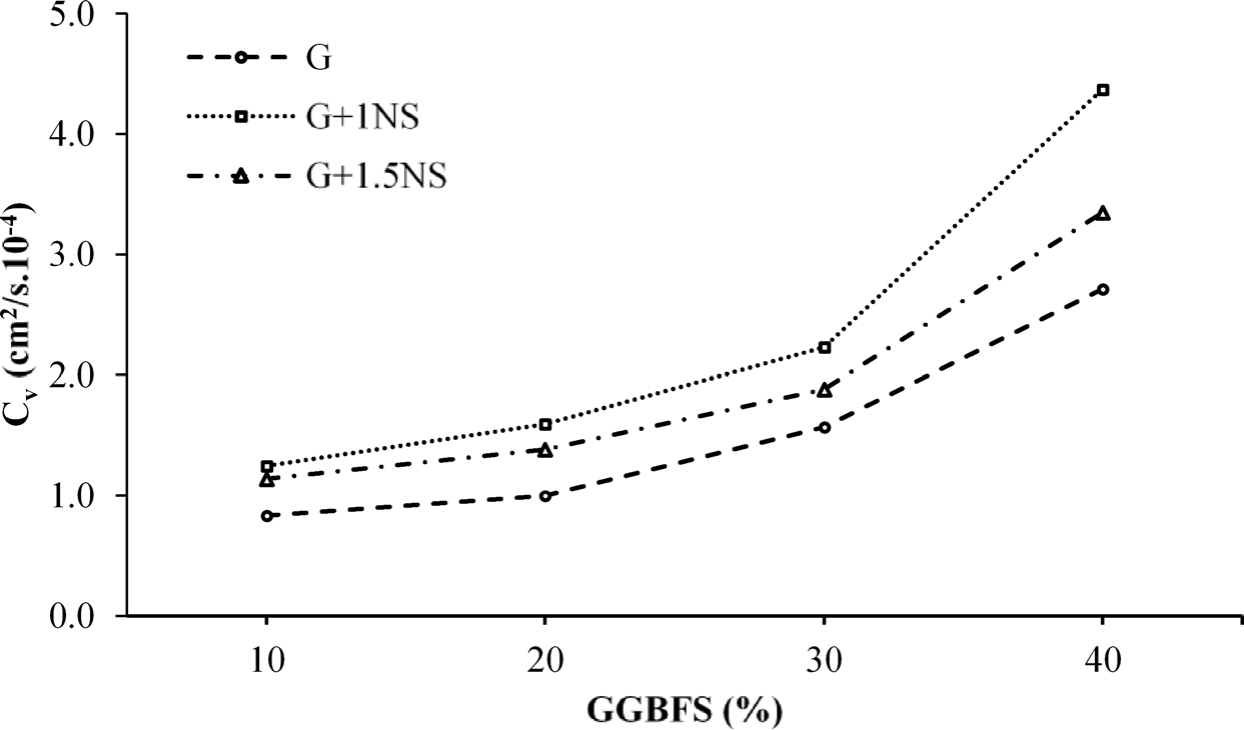
Variation of the c_v_ for GGBFS and NS stabilized LPHPC mixtures under 400 kPa.

The coefficient of consolidation (c_v_) reflects the rate at which excess pore water pressure dissipates during primary consolidation. For the untreated LPHPC, the cv value at the highest applied pressure (400 kPa) was found to be 0.489 cm^2^/s × 10^–4^. As illustrated in Fig. 9, c_v_ values increase with GGBFS content in all mixtures, suggesting that the use of GGBFS accelerates the consolidation process by improving drainage conditions and modifying soil structure. This effect becomes more pronounced at 30% and 40% GGBFS, indicating a threshold beyond which permeability and stiffness improvements contribute to faster consolidation.

The incorporation of NS further enhances the consolidation rate, particularly at 40% GGBFS, where the mixture with 1% NS exhibits the highest c_v_ value (Fig. 9). This improvement can be attributed to the micro-filling effect and pozzolanic activity of NS, which refine the soil matrix and promote faster dissipation of pore water pressure. However, the use of 1.5% NS yields slightly lower c_v_ values, possibly due to excessive fines reducing permeability. Therefore, the influence of NS on consolidation behavior is strongly dependent on its content and closely related to the initial structure and drainage capacity of the soil.

**Fig. 10:**
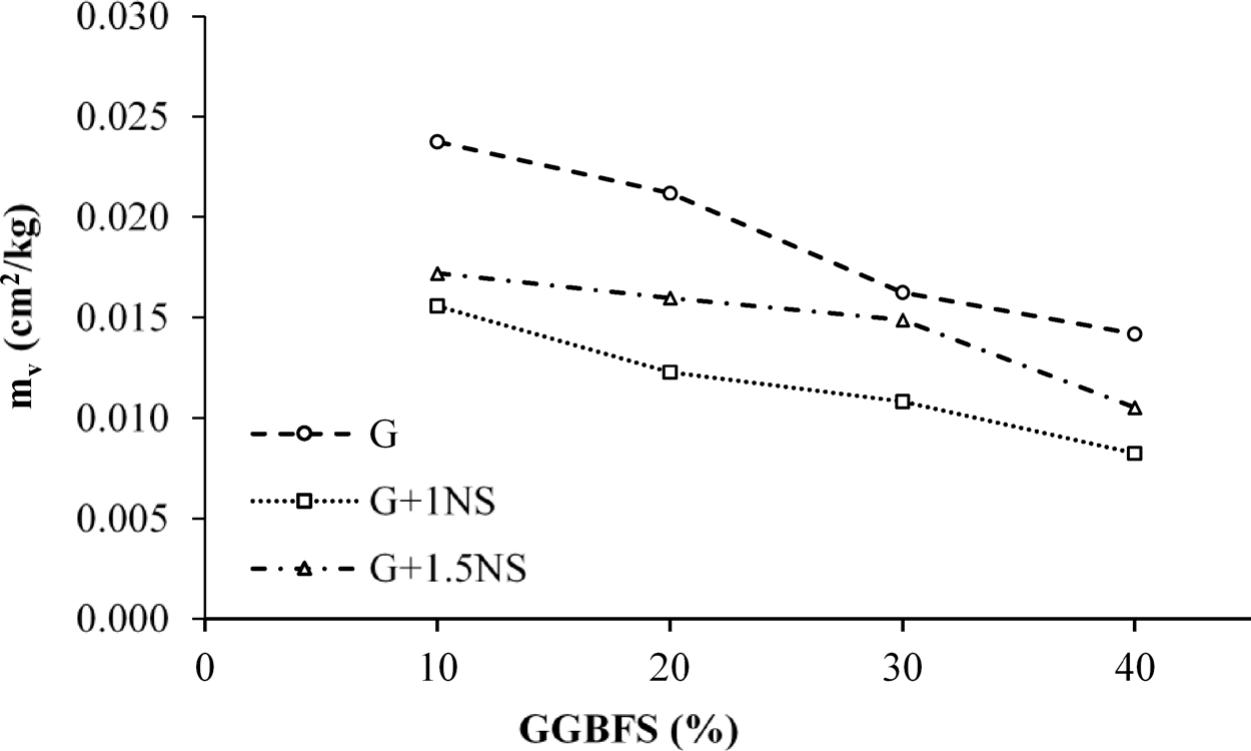
Variation of m_v_ in GGBFS and NS stabilized LPHPC mixtures under 400 kPa.

The coefficient of volume compressibility (m_v_) represents the magnitude of volume change that a soil undergoes per unit increase in effective stress during primary consolidation. The untreated LPHPC exhibited an m_v_ value of 0.0254 cm^2^/kg at 400 kPa, reflecting its volume change response to increased effective stress. Figure 10 illustrates the variation of the m_v_ values for LPHPC mixtures stabilized with GGBFS and NS. A general decreasing trend is evident as the GGBFS content increases from 10 to 40%, indicating a reduction in the compressibility of the treated soil and an enhancement in stiffness due to stabilization. Among the tested mixtures, the G + 1NS series exhibited the lowest m_v_ values at all GGBFS contents, confirming the beneficial effects of NS in reducing soil compressibility through micro-filling action and pozzolanic reactions. Conversely, the G + 1.5NS series showed slightly higher m_v_ values than G + 1NS, particularly at higher GGBFS contents, suggesting a possible threshold beyond which additional NS may not contribute further to performance improvement.

**Fig. 11:**
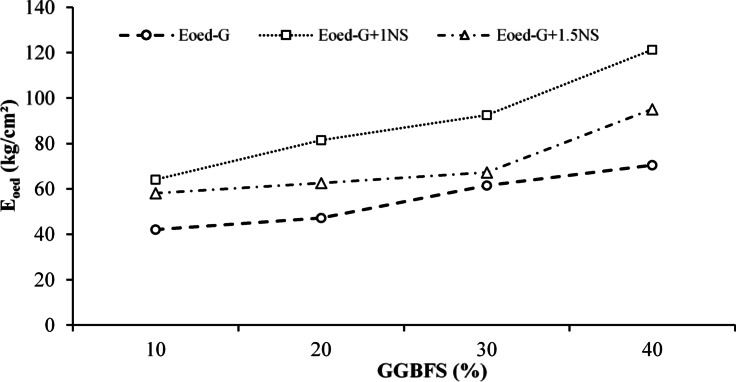
Variation of E_oed_ for GGBFS and NS stabilized LPHPC mixtures under 400 kPa.

The oedometric modulus (E_oed_) represents the stiffness of the soil during one-dimensional consolidation, indicating its resistance to vertical deformation under loading. To evaluate the consolidation-related stiffness behavior of the stabilized LPHPC mixtures, the variations in E_oed_ at an applied pressure level of 400 kPa were examined. These variations for LPHPC mixtures stabilized with different GGBFS contents and NS additions are illustrated in Fig. 11. The plotted lines represent E_oed_ values obtained directly from one-dimensional consolidation tests. The consistent increase in E_oed_ with GGBFS content, particularly in mixtures containing 1% NS, confirms the combined effectiveness of GGBFS and NS in enhancing the consolidation-related stiffness of LPHPC. Although a slight improvement was also observed for mixtures containing 1.5% NS, the enhancement was less pronounced compared to those with 1% NS, suggesting that the beneficial effect of NS on consolidation-related stiffness may level off beyond a certain NS content.

**Fig. 12:**
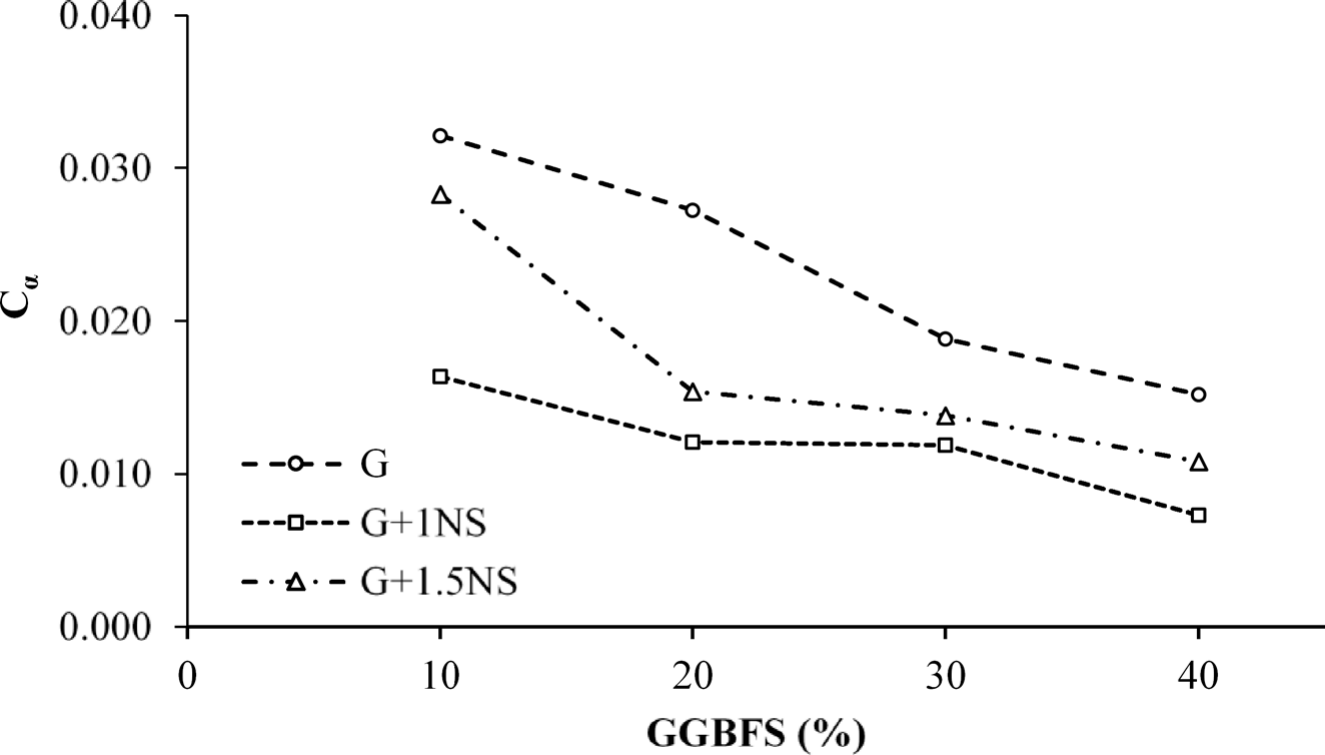
Variation in C_α_ of LPHPC Mixtures Stabilized with GGBFS and NS under 400 kPa.

The secondary compression index (C_α_), which characterizes the long-term compressibility of soil after the dissipation of excess pore water pressure, exhibited a decreasing trend with increasing GGBFS content across all mixtures (Fig. 12). This behavior indicates that the inclusion of GGBFS, particularly at higher ratios, contributes to the development of a more stable and less time-dependent soil structure. In mixtures containing 1% NS, C_α_ values remained consistently low, ranging from approximately 0.010 to 0.013, confirming the stabilizing role of GGBFS combined with NS in reducing creep-related deformations. Mixtures containing 1.5% NS exhibited relatively higher C_α_ values at lower GGBFS contents (10–20%), with values reaching approximately 0.030 at 10% GGBFS. This suggests that increased NS content may initially hinder structural densification due to increased water retention or delayed pozzolanic reactions. However, as the GGBFS content increased to 30% and 40%, the C_α_ values of these mixtures decreased markedly and converged with those of the mixtures containing 1% NS, indicating that higher GGBFS content can compensate for the adverse effects of elevated NS content.

Overall, the consolidation behavior of the stabilized LPHPC can be consistently interpreted through the combined evaluation of the consolidation parameters. The reductions observed in C_c_, C_s_, and m_v_ indicate a pronounced decrease in primary compressibility and recompression tendency, reflecting improved resistance to volumetric deformation. Concurrently, the increase in c_v_ values demonstrates an acceleration of the consolidation process, implying more efficient dissipation of excess pore water pressure. The corresponding increase in E_oed_ further confirms the enhancement of consolidation-related stiffness. In addition, the generally lower c_α_ values observed in stabilized mixtures suggest a reduction in time-dependent secondary compression. When considered collectively, these parameters provide a coherent assessment of settlement magnitude, consolidation rate, and long-term deformation behavior, supporting the effectiveness of GGBFS–NS stabilization from a practical consolidation perspective.

### Swell test

**Fig. 13:**
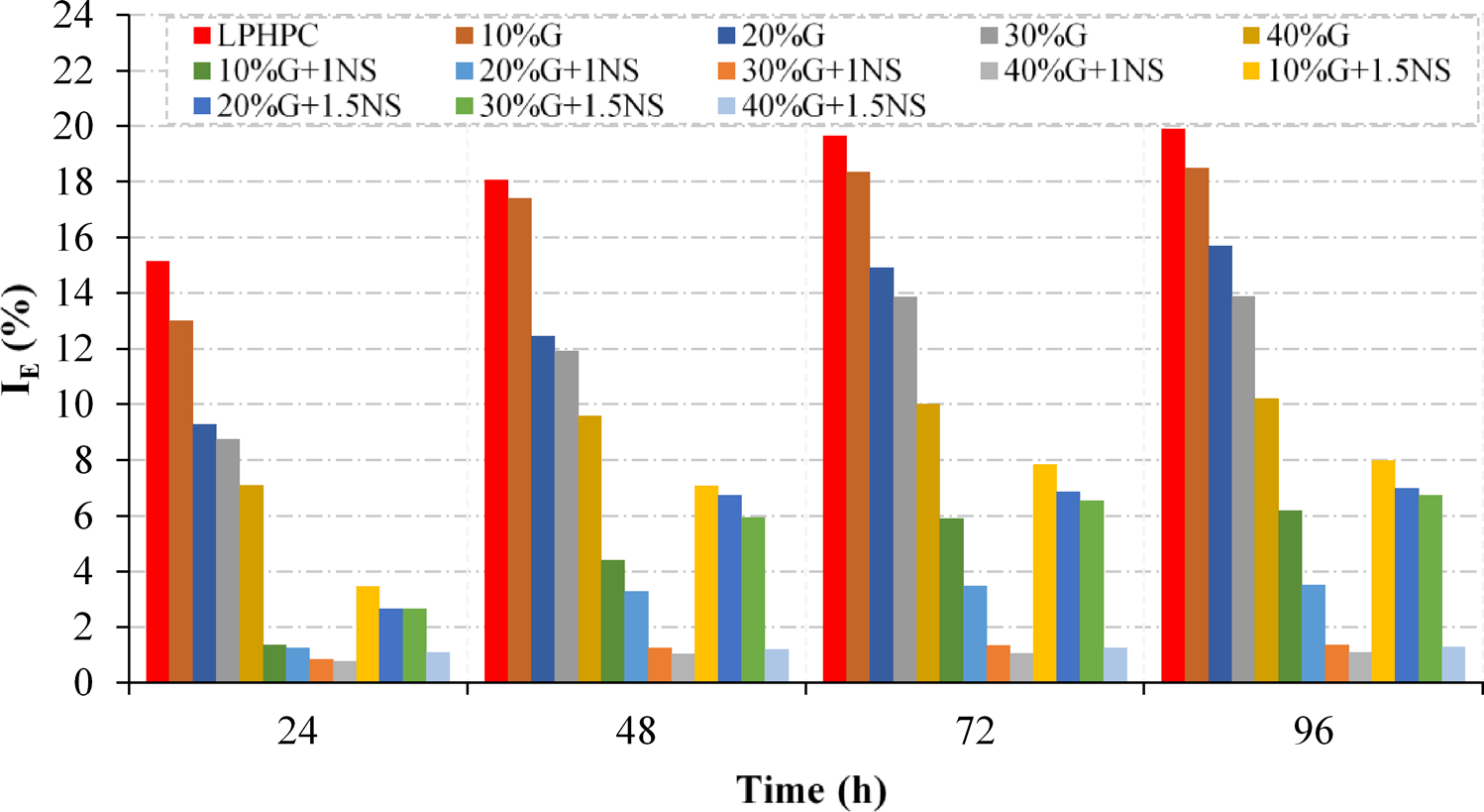
Time-dependent variation of the I_E_ for LPHPC stabilized with varying of GGBFS and NS.

The swell behavior of LPHPC stabilized with varying proportions of GGBFS and NS was assessed through one-dimensional swell tests. The influence of GGBFS and NS on the swell potential of the stabilized samples was evaluated by determining the expansion index (I_E_), in accordance with the primary swell criterion described by Day^56^. The variation of the I_E_ of LPHPC samples stabilized with different proportions of GGBFS and NS with time is presented in Fig. 13. The untreated LPHPC exhibited the highest I_E_ values at all curing times, indicating a strong swelling potential. The inclusion of GGBFS led to a notable reduction in I_E_, with the effect becoming more pronounced as the GGBFS content increased. Furthermore, the addition of NS (both 1% and 1.5%) further decreased the swell potential, particularly in mixtures containing 30% and 40% GGBFS. When the corresponding mixtures are compared, the combined use of GGBFS and NS is associated with an additional reduction of approximately 65% in I_E_ relative to mixtures stabilized with GGBFS alone, indicating improved volumetric stability over time. Overall, the results highlight that increasing the content of GGBFS and NS effectively mitigates the expansive behavior of the LPHPC, with the most significant improvement observed in mixtures with 40% GGBFS and NS (Fig. 13). Beyond the observed reduction in swelling indices, the mechanisms governing this behavior can be interpreted in terms of the stabilization processes induced by GGBFS and NS. The incorporation of calcium-rich GGBFS facilitates cation exchange on clay mineral surfaces, which reduces the thickness of the diffuse double layer and limits water-induced expansion. In parallel, the formation of gel-like reaction products contributes to fabric rearrangement and improved interparticle bonding, thereby restricting the separation and expansion of clay platelets upon wetting. The presence of NS further supports these mechanisms by promoting a denser soil fabric, leading to reduced swelling potential.

### Microstructural evaluation

The microstructural characteristics of stabilized LPHPC mixtures were investigated using scanning electron microscopy (SEM), energy-dispersive X-ray spectroscopy (EDS), and X-ray diffraction (XRD) analyses conducted on samples obtained after the completion of the one-dimensional consolidation tests. These analyses provided valuable insights into the microstructural evolution and elemental distribution within the LPHPC matrices treated with GGBFS and NS.

**Fig. 14:**
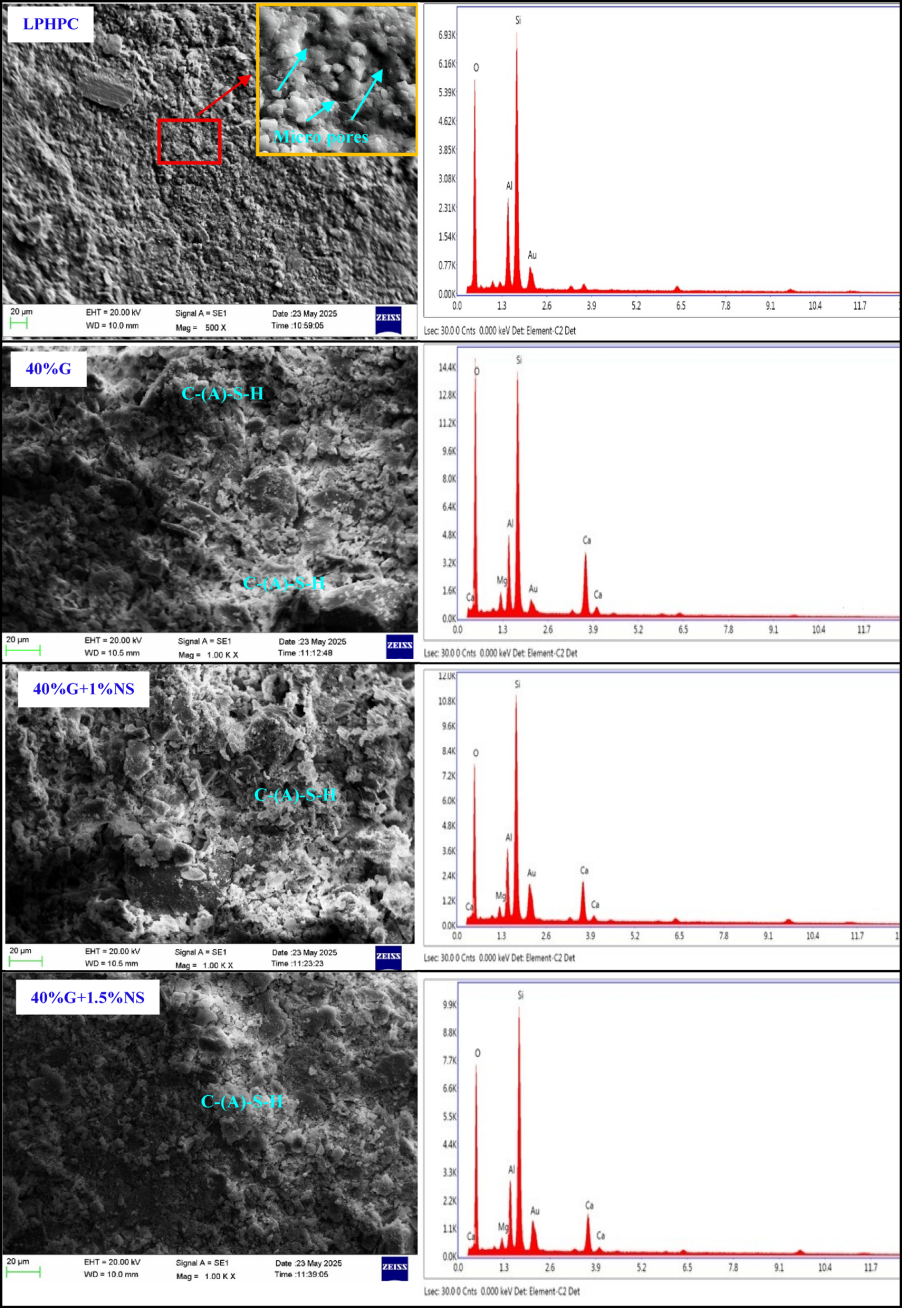
SEM and EDS results of untreated LPHPC and LPHPC stabilized with 40% GGBFS with and without NS.

The untreated LPHPC matrix exhibited a loose and porous structure with irregular particle contacts and an elemental composition primarily dominated by Si, O, and Al, which is consistent with the mineralogical characteristics of the raw clay material (Fig. 14). Upon stabilization with 40% GGBFS, the LPHPC matrix exhibited a denser and more cohesive structure, which was further reflected by the emergence of distinct calcium and magnesium peaks in the EDS spectra (Fig. 14). The observed microstructural densification and gel-like matrix development are consistent with possible molecular-scale chemical interactions. Recent molecular dynamics studies have suggested that moderate Ca^2+^ incorporation into aluminosilicate gel networks may facilitate framework rearrangement and favor the development of more polymerized and compact gel structures. Within this context, Ca^2+^ has been shown to contribute to charge compensation and bridging effects at the molecular scale, which can support enhanced network connectivity without implying direct phase identification or atomistic equivalence to experimental systems^57^.

The incorporation of 1% NS into the 40% GGBFS-stabilized mixture led to a further refinement of the microstructure, as evidenced by a denser and more homogeneous matrix with a greater extent of gel-like regions partially filling interparticle voids (Fig. 14). In these regions, EDS spectra show increased signals associated with Si, Al, and Ca. Consistent with common practice in microstructural interpretation, these regions are herein denoted as C–(A)–S–H type gel-like products based on their elemental composition and morphology, showing similarities to the gel structures typically associated with cement hydration products; however, this designation is employed for descriptive clarity and should be interpreted with appropriate caution regarding phase identification^58^.

**Fig. 15:**
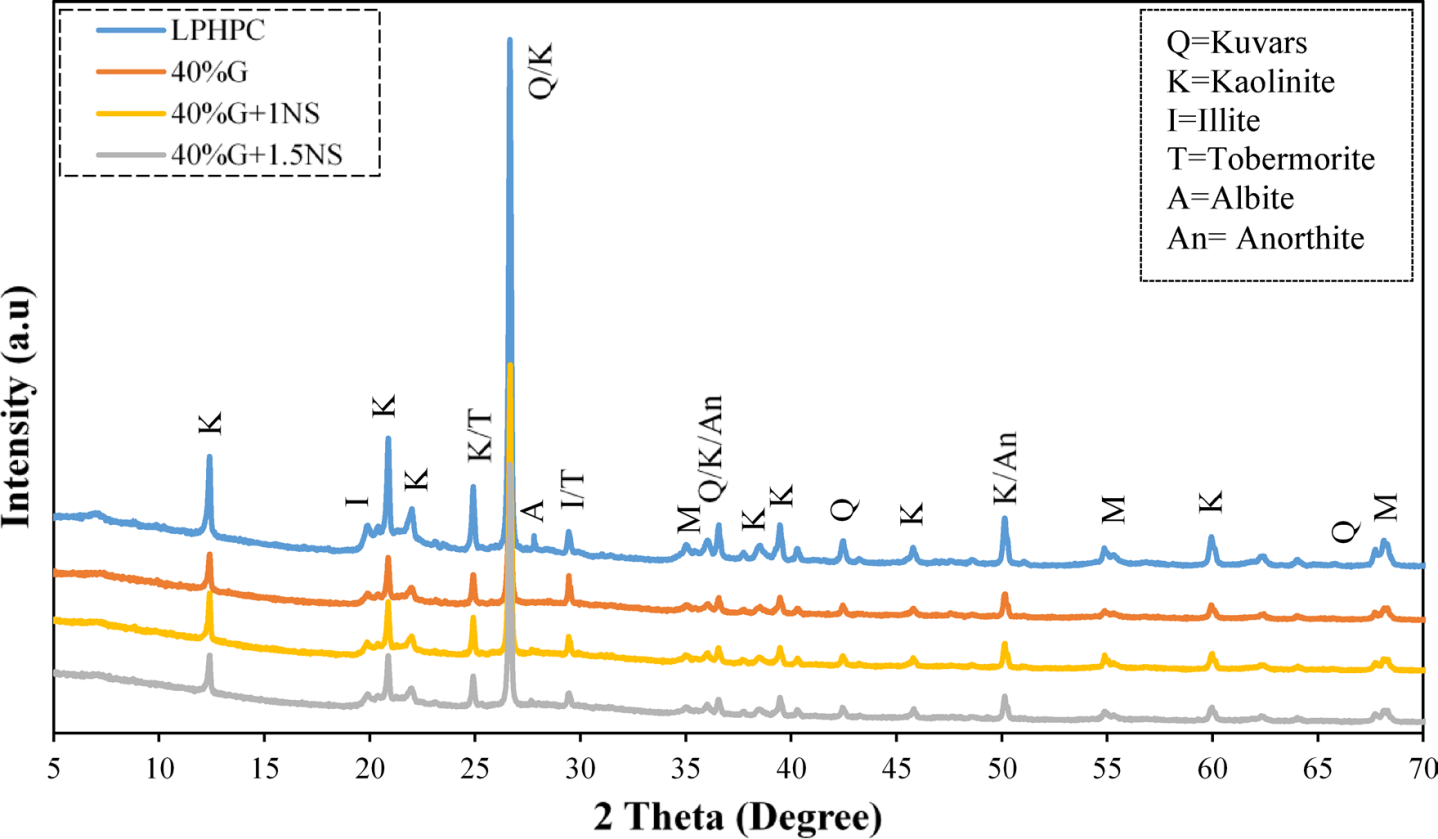
XRD patterns of untreated LPHPC and LPHPC stabilized with 40% GGBFS with and without NS.

Consistent with the mineralogical framework identified in Fig. 3, the XRD patterns of the stabilized mixtures show reflections associated with clay minerals such as kaolinite (K), quartz (Q), illite (I), montmorillonite (M), anorthite (An) and albite (A). Weak reflections that may be associated with tobermorite are also observed. From an XRD perspective, these features are reflected in Fig. 15 by the attenuation of clay-related crystalline peaks and a broader background hump, generally associated with partial amorphization and the development of poorly crystalline reaction products rather than distinct crystalline gel phases. Increasing the NS content to 1.5% was associated with a modest degree of additional local densification, as suggested by a partial reduction in visible voids and the emergence of more compact regions in the SEM images (Fig. 14). These locally compact domains, which are similarly enriched in Si, Ca, and Al according to EDS analysis, are described as C–(A)–S–H type gel-like regions based on their elemental composition and morphological features. Within this microstructural and mineralogical framework, the gel-like regions described as C–(A)–S–H type product can be considered structurally related to calcium silicate hydrate phase commonly reported in cementitious and alkali-activated systems. Crystalline calcium silicate hydrate named as tobermorite is often regarded as idealized or more ordered structural analogues of C–S–H type gel^59^. Accordingly, the weak or overlapping reflections tentatively attributed to tobermorite in the XRD patterns (Fig. 15) are interpreted as indicative of locally ordered domains within an otherwise poorly crystalline C–(A)–S–H type reaction matrix, rather than evidence of extensive crystalline phase formation. From a compositional perspective, the Ca–Si–Al chemistry of the present GGBFS–NS system can be interpreted using a ratio-informed framework based on the initial oxide compositions of the mixtures. Based on XRF-derived oxide contents and mixture proportions, the 40% GGBFS-stabilized LPHPC mixtures exhibit effective initial molar CaO/Al_2_O_3_ and SiO_2_/Al_2_O_3_ ratios of approximately 2.1 and 7.0 respectively. Recent studies have demonstrated that variations in these initial oxide ratios play a key role in governing reaction pathways, gel chemistry, and microstructural evolution in slag/fly ash-based geopolymer-stabilized clays. Moreover, studies considering clay mineralogy suggest that the effects of ratio-controlled chemistry depend on mineral dissolution and the participation of clay phases in gel formation. Accordingly, the ratio ranges identified in this study offer a chemically consistent basis for interpreting the SEM, EDS and XRD observations^60,61^.

## Conclusions

This study presents a comprehensive assessment of the influence of GGBFS and NS on LPHPC composed of 25% Na-bentonite and 75% kaolinite, based on a systematic evaluation of compressibility, consolidation response, swelling behavior, and microstructural characteristics. The stabilization design incorporated GGBFS at contents ranging from 10 to 40% by dry weight of soil as the primary additive, while NS at 1% and 1.5% was introduced exclusively in binary combination with GGBFS. An experimental program, including Atterberg limit tests, standard Proctor compaction, one-dimensional consolidation, swell tests, and microstructural analyses (SEM, EDS and XRD), was employed. Based on the experimental outcomes, the key findings of this investigation are summarized below.

1. The incorporation of GGBFS and NS significantly reduced the plasticity and consistency limits of the untreated clay, reflecting enhanced workability and lower swelling potential. These reductions in liquid limit and plasticity index are attributed to physicochemical interactions between the additives and clay minerals, including cation exchange and initial gel-like formation.
2. GGBFS addition resulted in a gradual decrease in MDD, which is associated with changes in compaction behavior and efficiency induced by slag incorporation, as evidenced by the corresponding MDD–OMC trends. However, at high slag contents, the benefits of increased NS content diminished, suggesting a potential upper limit for effective nanoparticle incorporation for the tested soil system.
3. One-dimensional consolidation tests demonstrated that the incorporation of GGBFS, particularly in combination with NS, markedly improved consolidation behavior of LPHPC. Progressive reductions in the C_c_ and m_v_ were observed with increasing stabilizer content, while the coefficient of consolidation and stiffness parameters showed a consistent increase. These improvements were attributed to microstructural refinement, enhanced pozzolanic reactivity, and improved interparticle bonding.
4. Increasing GGBFS content led to an improvement in secondary compression behavior. The addition of 1% NS further reduced post-consolidation deformation by enhancing gel-like bonding and particle packing. In contrast, 1.5% NS caused a slight increase in secondary compression at lower GGBFS levels, likely due to delayed pozzolanic activity, an effect that diminished with higher slag contents.
5. The swelling potential of LPHPC was substantially reduced through GGBFS based stabilization, and this effect was further enhanced by the addition of NS. Reductions in the I_E_ across all treated mixtures reflect improved soil fabric densification and the development of gel-like reaction products, leading to enhanced volumetric stability. While NS complemented the expansive clay mitigation provided by GGBFS, higher contents showed slightly reduced effectiveness particularly at lower slag levels likely due to excessive fines disrupting water distribution and pore structure.
6. Variations in clay mineralogy, including differences in surface activity and cation exchange capacity, as well as pore-water chemistry, may influence nanoparticle dispersion, reactivity, and water demand, thereby altering the optimal NS content. In this context, the optimal NS content identified in the present study should be regarded as soil-specific; while 1% NS was found to be the most effective for the investigated high plasticity clay, different soil systems may exhibit different optimal NS contents.
7. SEM–EDS and XRD analyses demonstrated that the combined use of GGBFS and NS promoted microstructural densification and the development of gel-like reaction products, as evidenced by SEM–EDS observations and supported by XRD trends. While the incorporation of NS contributed to further microstructural densification, excessive NS contents resulted in a less uniform distribution of gel-like domains, consistent with the plateau observed in compressibility and consolidation-related response.
8. Natural expansive clays may exhibit more complex behavior due to mixed clay minerals, organic matter, and pore-water chemistry; therefore, the findings of this study are intended to provide mechanistic insight, and extrapolation to natural expansive soils should be made with caution.

## Data Availability

All data generated or analyzed during this study are included in this published article.
